# MOFSynth: A Computational Tool toward Synthetic Likelihood Predictions of MOFs

**DOI:** 10.1021/acs.jcim.4c01298

**Published:** 2024-10-31

**Authors:** Charalampos G. Livas, Pantelis N. Trikalitis, George E. Froudakis

**Affiliations:** Department of Chemistry, University of Crete, Heraklion 71003, Greece

## Abstract

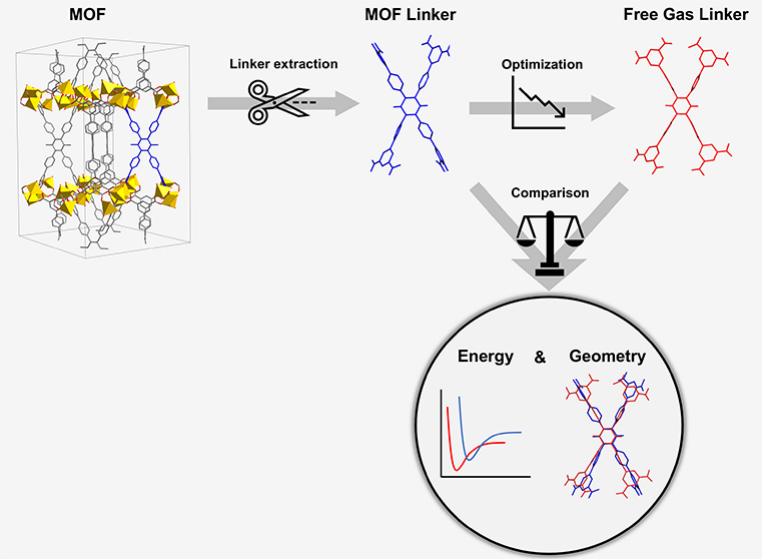

In the past decade, high-throughput computational studies of materials have increased significantly mainly due to advances in computer capabilities and have attracted a great deal of interest. In the field of metal–organic frameworks (MOFs), over a million hypothetical MOFs have been designed in silico, yet only a small fraction of these have been synthesized. For validating the computational-hypothetical results and accelerating the progress in the field, there is a pressing need for distinguishing MOFs that are more likely to be synthesized for real-life applications. This study presents a comprehensive investigation into the synthesizability likelihood of MOFs, utilizing a novel computational approach based on the disparities in energy and geometry between the linker conformation within the MOF structure and its isolated, free-gas state since both of these have been proven to be critical factors influencing MOF synthesis. Our user-friendly tool streamlines synthesizability evaluation, requiring minimal expertise in computational chemistry. By deconstructing over 40,000 MOFs from databases, including QMOF, CoRE MOF, and ToBaCCo, we analyze key parameters defining the linker strain within the MOF unit cell. Our results indicate that QMOF and CoRE MOF contain more promising candidates for synthesis, while ToBaCCo exhibits a relatively poor synthesizability likelihood due to unoptimized materials. Through extensive analysis, we identify optimal linker candidates for highly synthesizable MOFs. Consistent trends in energy distribution across databases that are confirmed by high Pearson and Spearman coefficients suggest the potential for omitting optimization calculations, significantly reducing computational costs. This study underscores the importance of linker deformation and energy disparities and enhances our understanding of synthetic accessibility in MOF research, offering valuable insights for future advancements in the field.

## Introduction

Reticular chemistry, the scientific approach of combining well-defined molecular building blocks in terms of size, shape, and connectivity to create extended, open-framework structures, provides chemists and materials scientists a versatile toolkit for the development of advanced materials suitable for addressing various challenges.^1^ Metal–organic frameworks (MOFs) are a great example of the successful implementation of reticular chemistry and, therefore, have captured significant interest in targeting tailor-made materials. MOFs represent a distinctive class of nanoporous materials characterized by precisely defined pore shapes, sizes, and chemistry. These materials exhibit exceptional physical properties, including extremely high porosity (up to 90% free volume), low density, and a large surface area. Their construction is modular, achieved through the cooperative self-assembly of inorganic units (comprising metal ions or clusters) and organic linkers, resulting in diverse framework topologies and creating a vast combinatorial design space. The strategic selection of inorganic building blocks and organic linkers allows for the tailored design of MOFs optimized for specific environmental, health, and energy applications.^2−6^ Despite this potential, the central challenge lies in pinpointing the most optimal and feasible combination of MOF building blocks and their configurations within this practically limitless design space.

Despite the promise of metal–organic frameworks in various applications, their experimental study poses considerable challenges. Traditional methods for synthesizing MOFs often require intricate control over reaction conditions and necessitate time-consuming trial-and-error processes. The sheer complexity and diversity of potential MOF structures exacerbate these challenges, making it difficult to predict the synthesizability of certain MOFs, their optimal synthesis routes, and finally the measurement of the desired properties.

Recognizing these difficulties, there is a compelling need for advanced in silico tools. Computational approaches that involve ab initio calculations, classical simulations, and machine learning offer a systematic and efficient means to explore the expansive MOF design space and accelerate the discovery of new and desirable MOFs. Databases containing an immense number of metal–organic frameworks, potentially reaching into the trillions, can be studied in order to aid the scientific community in identifying desired materials within a fraction of the time needed for experimental analysis.

While numerous studies employing high-throughput computational screening (HTCS) methods have gained considerable attention, they often overlook a crucial parameter: the synthesizability of the proposed materials. Although these investigations excel in identifying top-performing candidates for various applications within expansive databases, they commonly neglect a fundamental aspect that will lead to the implementation of these materials in the real world.

A noteworthy example is the computation-ready, experimental (CoRE) MOF database.^7,8^ Chung et al. mention that their main target is to allow a HTCS screening of numerous materials, but the optimal ones found need to be further analyzed for stability. They propose performing geometry optimization calculations and subsequently recomputing the desired properties. Many studies employ computational tools to scan through the CoRE MOF database, but they do not proceed to further support their findings.^9−13^ Simon et al.^9^ advocate for leveraging a materials genome approach to expedite the discovery of high-performance adsorbent materials, particularly focusing on metal–organic frameworks for natural gas storage in vehicles. They compiled methane uptake data from over 650,000 materials, including 5109 from the CoRE MOF database, but they neglected to study the synthesizability likelihood of the best-performing ones. Similarly, He et al.^13^ developed a strategy for screening high-performance bio-metal–organic frameworks (bio-MOFs) for oxygen (O_2_) separation from air, utilizing machine learning and molecular simulation techniques. They selected desired bio-MOFs from MOF databases using a binary decision tree method, characterized them using 15 descriptors, and employed a Random Forest (RF) algorithm to establish mapping between descriptors and target properties obtained from Grand canonical Monte Carlo (GCMC) simulations. Through high-throughput screening, they identified high-performance bio-MOFs for O_2_/N_2_ adsorption separation. However, they neglected to assess the synthesis ability of the identified high-performance bio-MOFs, focusing solely on their separation efficiency.

Numerous other studies that utilize other big databases acknowledge the fact that they do not consider the practical feasibility of top-performing materials synthesis. Lee et al.^14^ propose a systematic strategy utilizing machine learning and evolutionary algorithms to sift through an extensive set of over 100 trillion possible metal–organic frameworks. This strategy addresses the limitations of traditional computational screening approaches, which often employ a brute-force strategy and are restricted to an initial set of materials. Their approach identifies 964 MOFs with methane working capacities exceeding 200 cm^3^/cm^3^, including 96 surpassing the world record of 208 cm^3^/cm^3^. Notably, the study acknowledges the challenges of experimental synthesis and omits top-performing MOFs from consideration due to perceived difficulty, emphasizing the need for a more practical approach to material discovery. In another study by Ahmed and Siegel^15^ machine learning is leveraged to predict hydrogen capacities for a diverse set of 918,734 MOFs sourced from 19 databases. Using only 7 structural features, the model identifies 8282 MOFs with the potential to surpass state-of-the-art materials, characterized by low densities, high surface areas, void fractions, and pore volumes. However, the study concludes that limitations exist, particularly in the synthesis feasibility of some high-capacity MOFs, emphasizing the challenges of experimental realization and the potential for future advancements in synthesis techniques to overcome these barriers.

One way to make traditional HTCS more effective in finding new MOFs is to implement a rapid and easy-to-use protocol to predict the likelihood of MOF synthesizability. Even though recent experiments^16−18^ have provided useful insights into MOF formation, it is still unclear what exactly determines the outcome of the experimental synthesis.

The ability to bridge this gap between theoretical predictions and experimental realization is paramount for ensuring the viability and applicability of metal–organic frameworks in practical scenarios. Presently, decisions regarding the synthesis of top-performing candidates following HTCS studies rely on chemical intuition. Due to the expense of exploratory experiments, those efforts are typically directed toward MOFs that resemble previously synthesized ones. This practice may overlook truly exceptional candidates, impeding the effectiveness of MOF discovery and limiting the exploration of novel regions within the MOF space. This observation is further supported by the fact that approximately 50% of over 40,000 published articles regarding MOFs concentrate on just 30 cases as mentioned by Anderson and Gómez-Gualdrón.^19^

In addition, such an evaluation tool could function as an initial screening tool in HTCS investigations, enabling the exclusion of MOFs with a poor synthesis probability. This targeted strategy ensures efficient allocation of computational resources toward MOFs with higher potential for synthesis post-HTCS. The adoption of a synthetic likelihood criterion would allow reduction of the database, facilitating simulations with certain computational constraints.

Nevertheless, there is a noteworthy lack of studies that specifically concentrate on assessing the likelihood of synthesizability of porous materials. Jablonka et al.’s review^20^ covers principles of big-data science, including the selection of training sets, representation of materials in feature space, learning architectures, and evaluation strategies. Additionally, it explores the application of machine learning in various aspects of porous materials, such as stability and synthesis. Regarding the latter, a limited number of studies, focusing specifically on zeolites, are mentioned, revealing the relatively sparse research conducted in this area. According to this review, early works proposed that low framework energies are the distinguishing criterion for a high likelihood of synthesizability of zeolites, but this was quickly rejected with the discovery of high-energy ones. It was replaced by a “flexibility window”, which was later shown to be unreliable and replaced with criteria that focus on local interatomic distances. Perez et al.^21^ conducted a screening study that used a library of such energetic and structural criteria. Their work concludes by proposing the use of the overlap between the distribution of descriptors of experimental materials and those generated in silico as a metric to assess the feasibility of the materials. Nonetheless, this approach is too complex for individual handling and expediting the process, requiring expertise from a computational specialist and a plethora of experimental data. In another study, Anderson et al. introduced a crystallographic net rescaling algorithm to the topologically based crystal constructor code, ToBaCCo 3.0, facilitating the automated construction of MOFs with varying topologies.^19^ By computationally “synthesizing” isomorphic MOFs, the researchers demonstrated the significant influence of crystal topology on adsorption and mechanical properties. They evaluated the mechanical stability of a material through the Born stability criterion, highlighting the importance of the latter in identifying realistic targets for synthesis. Nevertheless, it is essential to note that calculating the elastic constants computationally, as is conventionally done with LAMMPS, involves intensive work. The same group conducted large-scale calculations of MOFs’ free energies on a diverse database of 8500 MOFs, recommending the use of Frenkel–Ladd (FL) path thermodynamic integration coupled with UFF4MOF for accurate estimation.^22^ They identified two potential criteria for identifying synthetically likely MOFs: a linear fit of free energies to the metal/linker atom ratio and selecting the MOF with the lowest-predicted free energy within the isomorphic series, highlighting the importance of thermodynamic stability in determining synthetic accessibility. However, this method does not provide a user-friendly program and necessitates extensive molecular dynamics simulations and expertise. A solid foundation and proficiency in computational chemistry are required to cope with the complexities of the calculations and ensure accurate and meaningful results.

In response, we propose that the disparities in energy and geometry between the organic linker conformation within the MOF structure and the conformation observed in its isolated, free-gas state are crucial factors in MOF synthesis as they represent the energetic barriers that must be overcome for successful formation, influencing the overall synthesizability of MOFs. It is noted that the selection of the organic linker in MOF synthesis is of paramount importance because the vast majority directs the in situ formation of the inorganic building blocks, and for this reason reticular synthesis approaches start with the selection of the linker in terms of size, shape, and number of coordination.^23^ Our approach requires minimal expertise and knowledge in computational chemistry and streamlines the process of synthesizability evaluation. The devised workflow involves the extraction of the MOF’s organic linker, followed by an optimization procedure of the ligand to attain its free conformation energy. We assess the synthesizability likelihood by analyzing geometric changes and the corresponding energy differences resulting from the optimization process. In the following sections, in addition to describing our code, we elaborate on the utilization of our model in distinctive cases. We present an extensive study on three databases, namely, Quantum MOF (QMOF),^24^ CoRE MOF,^7^ and ToBaCCo database^25^ to showcase the validity of the tool and its capabilities to handle big databases within a short time frame. The three aforementioned databases were strategically selected, as their generation followed different rules and methodologies. ToBaCCo is a hypothetical database in contrast to QMOF and CoRE MOF both containing experimental instances. In addition, QMOF’s entries are optimized, leading to the expectation that this database would contain candidates with easier synthesis and suitable for further experimental studies.

## Methodology

The initial step in the synthesizability evaluation involves the creation of a supercell, accomplished by multiplying the unit cell’s dimensions by a factor of 2. This expansion ensures that the MOF’s cell under investigation encompasses a complete linker, avoiding partial representations common in many unit cells due to symmetry operations. However, for larger CIF files that already meet this condition, the supercell creation is redundant. Therefore, we introduced an optional feature to prevent the creation of a supercell if all cell dimensions exceed a user-specified threshold, defined in Angstroms. This threshold can be set to any positive number that the user considers appropriate for their use case and is used to streamline the process and reduce the computational time. This crucial procedure is seamlessly executed using the pymatgen^26^ library. It is an open-source Python library renowned for its robustness in materials analysis.

**Figure 1:**
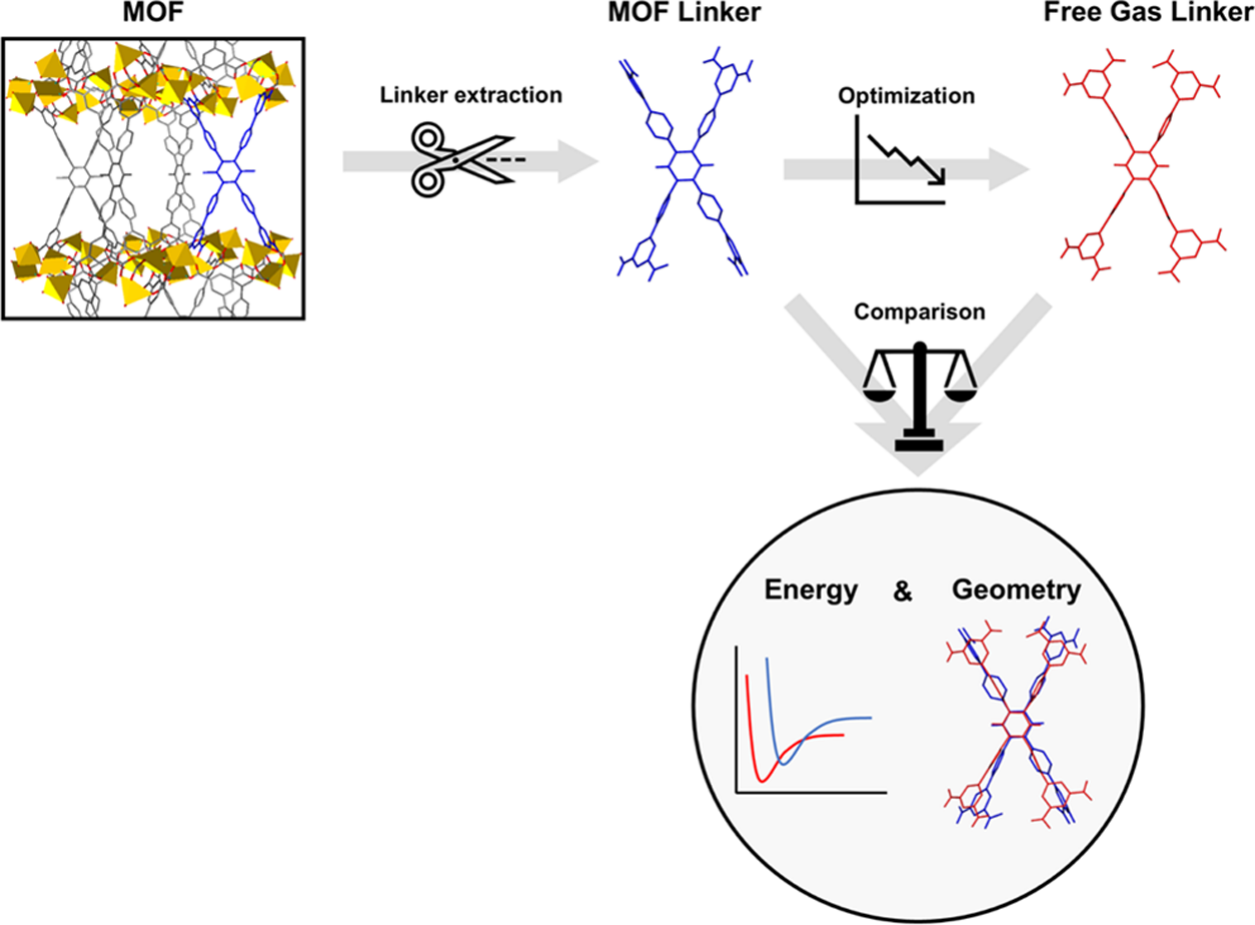
MOFSynth workflow: The first step involves linker extraction followed by optimization. The final step consists of comparing the original and optimized structures.

The subsequent step in the synthesizability evaluation, as seen in Figure 1, involves a fragmentation procedure, where the linkers of the previously created supercell are extracted into a separate file. To execute this task, the MOFid module is employed.^27^ Leveraging this algorithm enables the extraction of all linkers in CIF file format. This comprehensive extraction proves to be invaluable for the subsequent optimization stage and the correct representation of the linker.

In the next step, the above-mentioned CIF file undergoes parsing through Open Babel.^28^ Through utilization of the latter, the outcome is an XYZ file containing the coordinates of a single linker representative of the metal–organic framework under examination.

In the fourth step, we employed TURBOMOLE^29^ for both a single-point calculation and an energy optimization procedure. The calculations utilized the Universal Force Field (UFF),^30^ with the convergence criteria set at 10^–7^ atomic units and 10^–4^ Angstroms for energy and geometry cycles, respectively. The maximum displacement for a coordinate in a relax step was set to 0.30 atomic units, nqeq is zero so the partial charges were calculated only in the first cycle, and iterm was set to 111111 in order for the bond, angle, torsion, inversion, non-bonded van der Waals, and non-bonded electrostatic terms to be calculated. Finally, if the norm of the gradient is greater than 100, a deepest-descent step will be done, and if it is smaller than 10^–3^, no line-search step will be done after the Newton step. The outcome of this step yields two distinct conformations: one representing the initial state of the linker within the metal–organic framework and the other showcasing the optimized configuration. These conformations provide valuable insights into the structural changes and energy surface during the optimization process, contributing essential data for the overall assessment of the synthesizability.

The calculation of energy and geometrical changes was done in the last phase of our synthesizability assessment. Initially, linkers were grouped based on their SMILES code, which was extracted using RDKit.^31^ This was followed by a comparison of each group’s optimized energies and retention of the conformation with the lowest-optimized energy. The energy difference for each linker was determined by computing the disparity in its single-point energy and the lowest-optimization energy of its group. Furthermore, the quantification of geometric deformation was quantified with the root-mean-square deviation (RMSD). Regarding the latter, to ensure accuracy, the two monomers was subject to a recentering process and were rotated to achieve the true minimal RMSD. This step is pivotal because a straightforward calculation of the RMSD may yield irregular values. The RMSD computation employed the Kabsch algorithm,^32^ renowned for its efficacy in determining the optimal rotation matrix that minimizes the root-mean-square deviation between two sets of Cartesian coordinates. The utilization of an open-source program^33^ ensured the precision and reliability of these calculations.

As part of this project, we are releasing an open-source code on GitHub (https://github.com/frudakis-research-group/mofsynth), which includes an implementation of the MOFSynth procedure analyzed above. The use of open-source software for the MOFSynth scheme is advantageous. Users can inspect the source code of the underlying algorithms and the repositories that facilitate distribution of the code to the research community. In addition, we provide our tool in a web interface (https://mofsynth.website), which allows researchers with minimal computational knowledge to use our evaluation tool and extract information on MOFs of their choice.

## Results and Discussion

Our primary objective was to evaluate the performance of our code across a variety of databases with distinct characteristics and compositions that would allow us to assess the robustness and versatility of our methodology. We utilized the quantum MOF database,^24^ the computation-ready experimental metal–organic framework^7^ database, and the ToBaCCo database^25^ in order to compute the synthesizability likelihood of the contained entries. QMOF is a publicly available database of computed quantum-chemical properties for more than 20,000 experimentally synthesized MOFs. To our knowledge, this is one of the most accurate and notable databases in the literature. The CoRE MOF database incorporates over 14,000 porous, three-dimensional metal–organic framework structures. The updated version encompasses additional contributions from CoRE MOF users, data from the Cambridge Structural Database, and a Web of Science search. We studied the all solvent removed subset, which includes 10.143 CIFs with both free and bound solvents removed. Additionally, the ToBaCCo database includes 13,512 hypothetical MOFs of 41 distinct topologies and is extensively explored for numerous applications.

**Figure 2:**
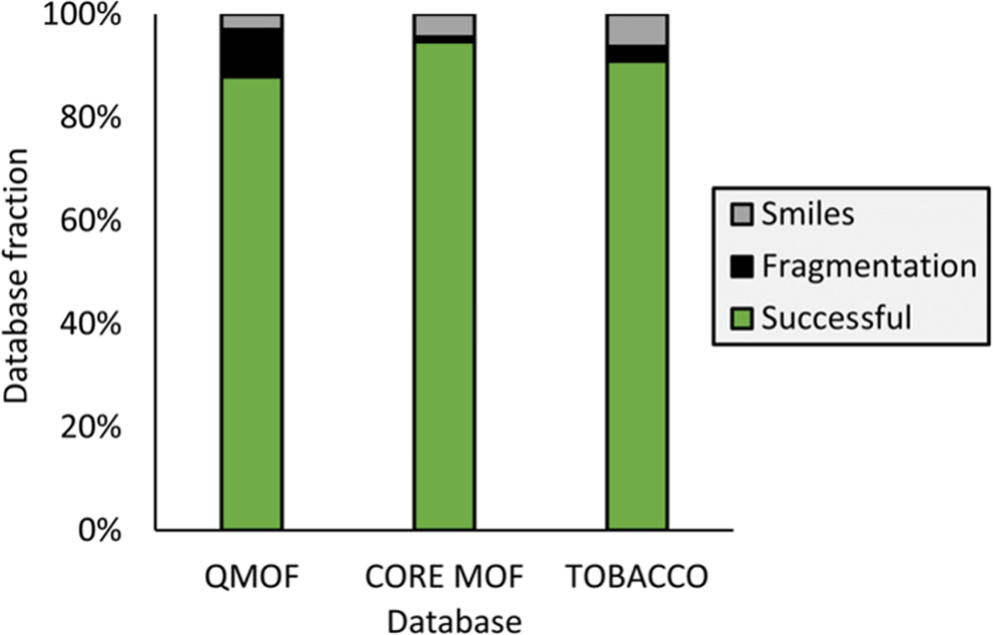
Success and failure rates in fragmentation and SMILES procedure.

As evident from Figure 2, our code effectively parsed the vast majority of instances. Specifically, the protocol was successfully employed for 17,910 MOFs out of the initial 20375 CIFs provided for QMOF, for 9608 instances originating from the CoRE MOF, and for 12287 instances of the ToBaCCO database. The remaining instances encountered issues during the fragmentation procedure and faced challenges in identifying the SMILES code. Our workflow shows great robustness and adaptability, showcasing its ability to analyze various types of CIFs with the majority of instances being successfully processed.

Upon analyzing the results, very few instances exhibited negative linker single-point energy, which is inconsistent with classical energy calculations. Furthermore, a few MOFs displayed exceedingly high energies (>500 kcal/mol), prompting their exclusion from subsequent visualizations for clarity.

**Figure 3:**
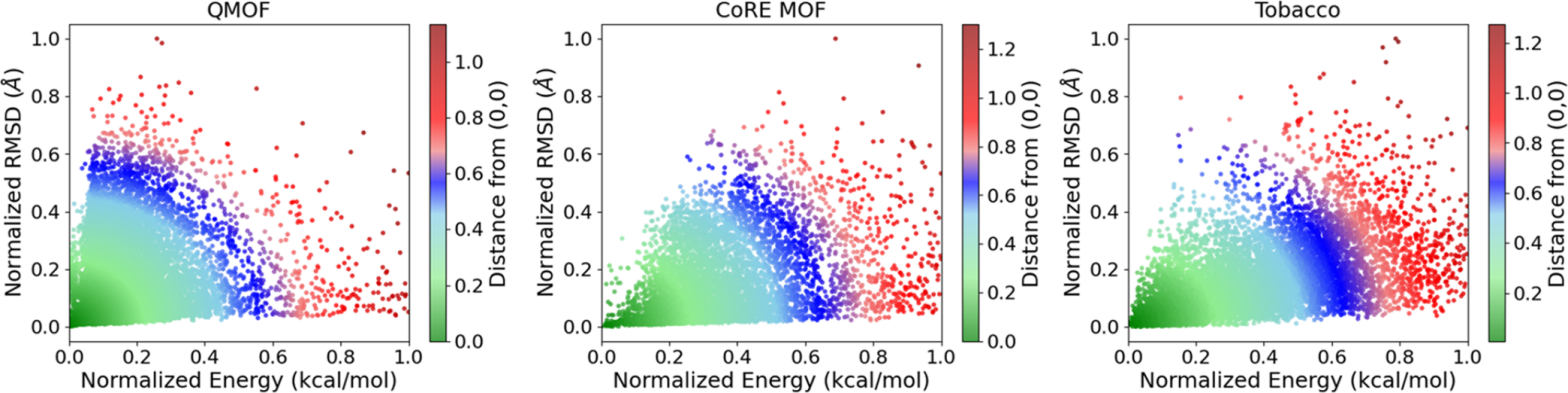
Scatter plot of normalized RMSD versus normalized Δ*E* values.

Figure 3 depicts the normalized root-mean-squared displacement (RMSD) along with the normalized energy difference (Δ*E*) of each database’s instances. A color gradient is applied to the scatter plots, indicating the distance of each instance from the (0,0) point. This coloring scheme serves as a metric for assessing the ease of synthesizing each material. Instances farther from the origin are less likely to be synthesized. Upon initial observation, as evidenced by the colormap range, CIFs of the QMOF database have on average lower-energy differences and are closer to the origin of the axis. The colormap ranges from 0 to 1 for the QMOF, while for the CoRE MOF and ToBaCCo, it spans from 0 to 1.2.

**Figure 4:**
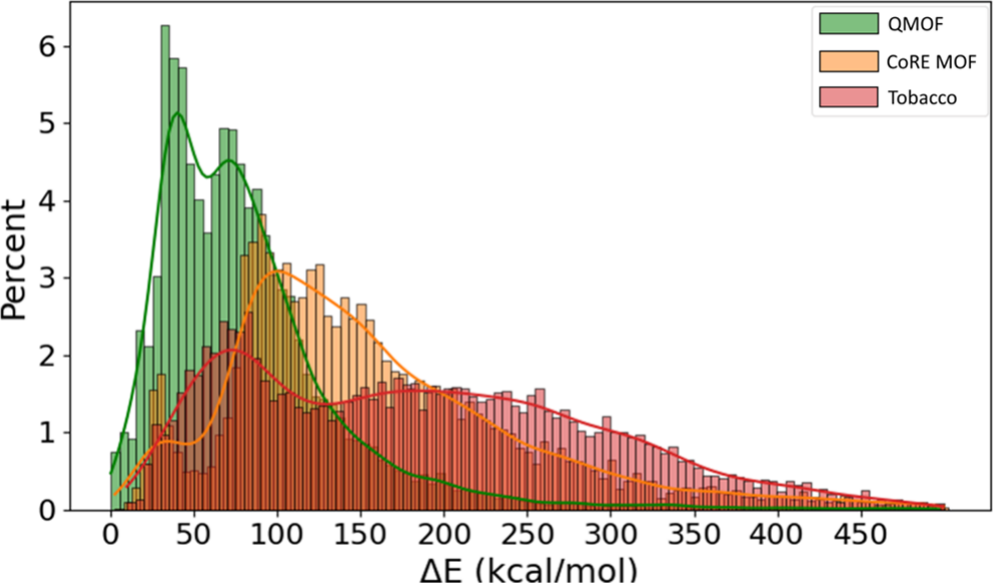
Percentage distribution of Δ*E* (kcal/mol).

An important distinction between the three databases under study is revealed in Figure 4, which shows the energy percentage distribution plot along with a kernel-density estimate plot. In the QMOF, the majority of cases are concentrated below 100 kcal/mol, with a peak at around 40 kcal/mol. On the contrary, the majority of MOFs of the CoRE MOF are clustered above the 100 kcal/mol mark, with a peak distribution around 85 kcal/mol. This observation suggests that QMOF contains more energetically stable instances, aligning with our initial expectations based on the inherent characteristics of the two databases. On the other hand, ToBaCCo’s distribution depicts slight peaks with the larger part of the database being evenly distributed along the energy axis up to the 250 kcal/mol mark where a gradual decline begins. This is a significant result, considering that the ToBaCCo database consists of hypothetical materials, as mentioned above. These materials are not optimized and may possess atypical properties, such as oversized bonds, which could impact their synthesizability.

**Figure 5:**
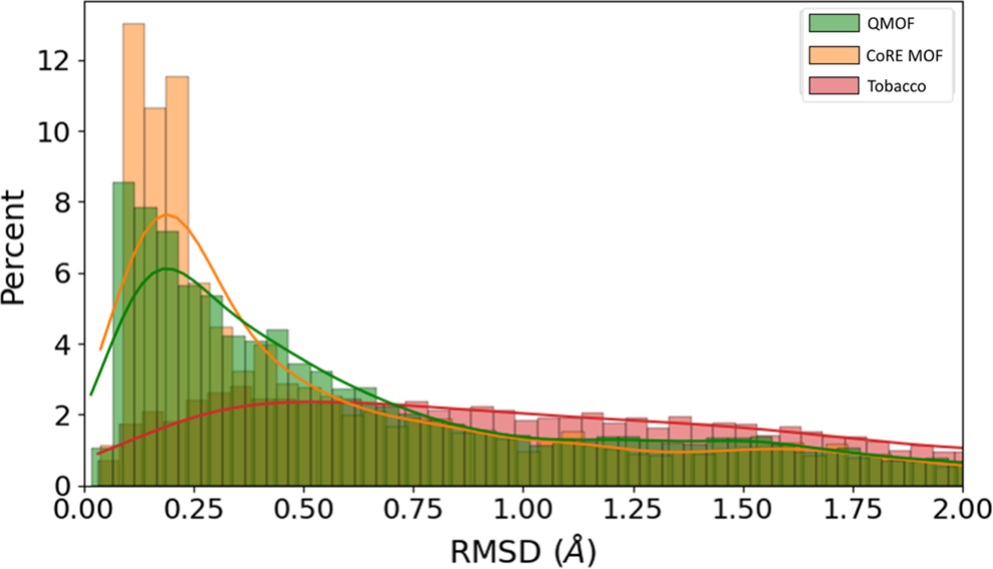
Percentage distribution of the root-mean-square deviation (Å).

Regarding the root-mean-square-deviation-distribution plot, which is depicted in Figure 5, similar trends are observed. We must note that for visualization purposes we have excluded RMSD values greater than 2 Å. Interestingly, both QMOF and CoRE MOF display a peak at small RMSD values, although QMOF’s peak is slightly lower. Consequently, QMOF tends to have a slightly higher number of MOFs in the following RMSD regions. However, this trend converges quickly at around 0.7 Å, where both databases exhibit comparable distributions once again. The distribution of hypothetical MOFs, once again, lacks a distinctive peak and showcases an even spread along the RMSD axis. This finding further supports our initial expectations that the hypothetical MOF database would comprise MOFs inclined toward greater deformation during their unit cell assembly.

**Figure 6:**
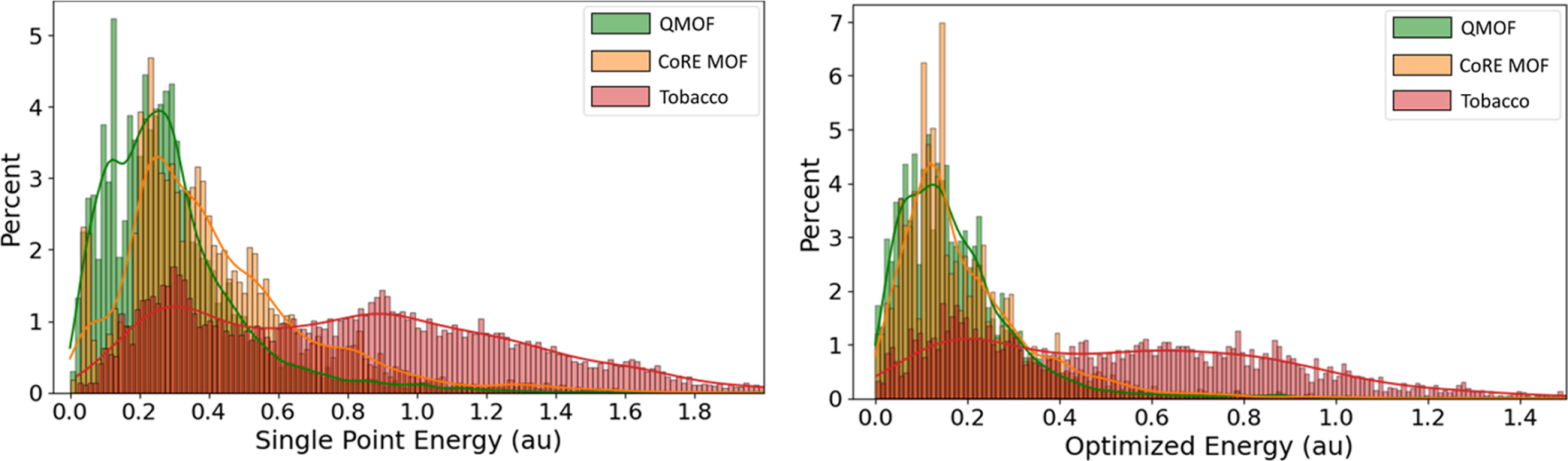
Percentage distribution of single-point energy (left) and optimized energy (right) in atomic units.

**Figure 7:**
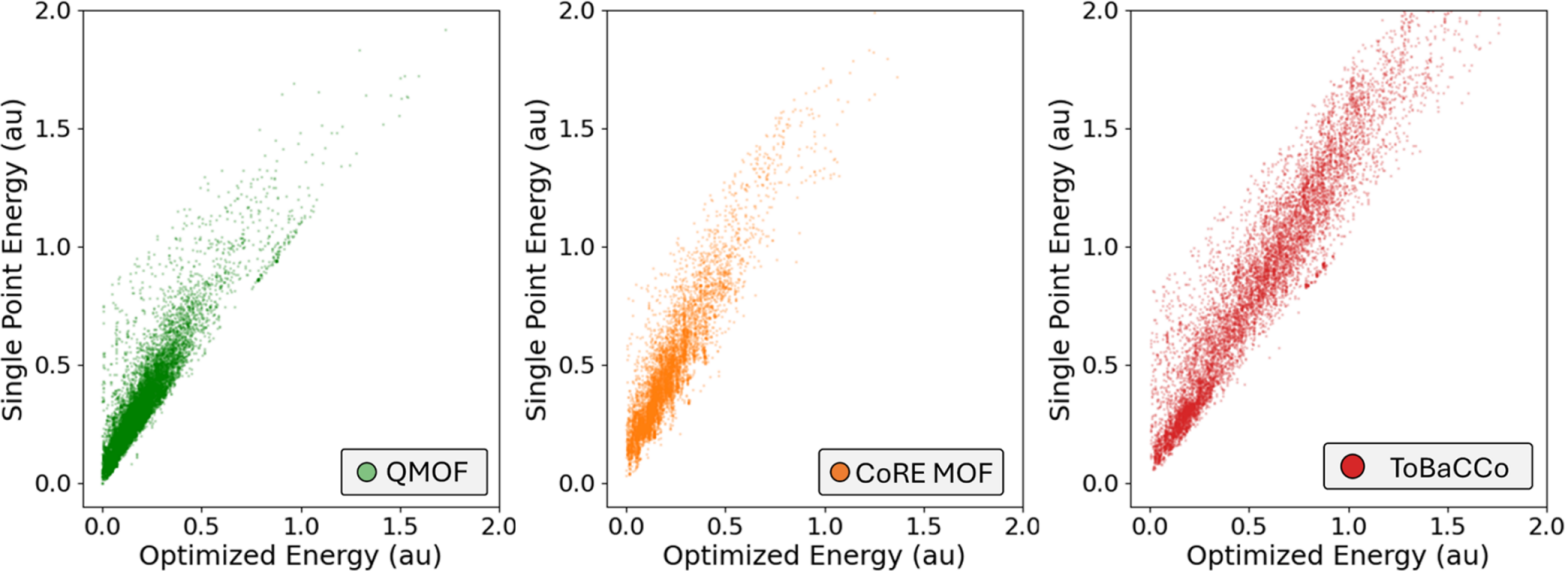
Scatter plot of single-point energies vs optimized energies of MOF linkers in atomic units.

In Figure 6, the distributions of the linker’s single-point (SP) and optimized (OPT) energies are depicted, revealing a comparable trend between the two. In both distributions, the QMOF and CoRE MOF show a higher concentration of materials in the low-energy region and experience a rapid decline in the higher-energy regions. On the other hand, TobBaCCo exhibits an even distribution pattern characterized by two minor peaks. The similarity between the two sets of data can be quantified by the Pearson (R) and Spearman (S) correlation coefficients. An R-value close to 1 indicates a strong linear correlation between SP and OPT energies, and an *S*-value close to 1 suggests that the ordering of MOFs by the two energies would yield similar results. In Figure 7, we see three distinguished scatter plots for all databases that show the SP vs OPT energy along with the point density. The linear relation is evident, especially through examination of the latter plot. The calculated Pearson and Spearman coefficients shown in Table 1 further support the argument that the two properties have a strong correlation. This implies that the linker-optimized energy could be estimated using its single-point energy, reducing the computational cost significantly.

**Table 1:** Pearson and Spearman Coefficient Values for the Three Databases under Study

Correlation	Pearson	Spearman
QMOF	0.90	0.89
CoRE MOF	0.87	0.85
ToBaCCo	0.95	0.92

**Figure 8:**
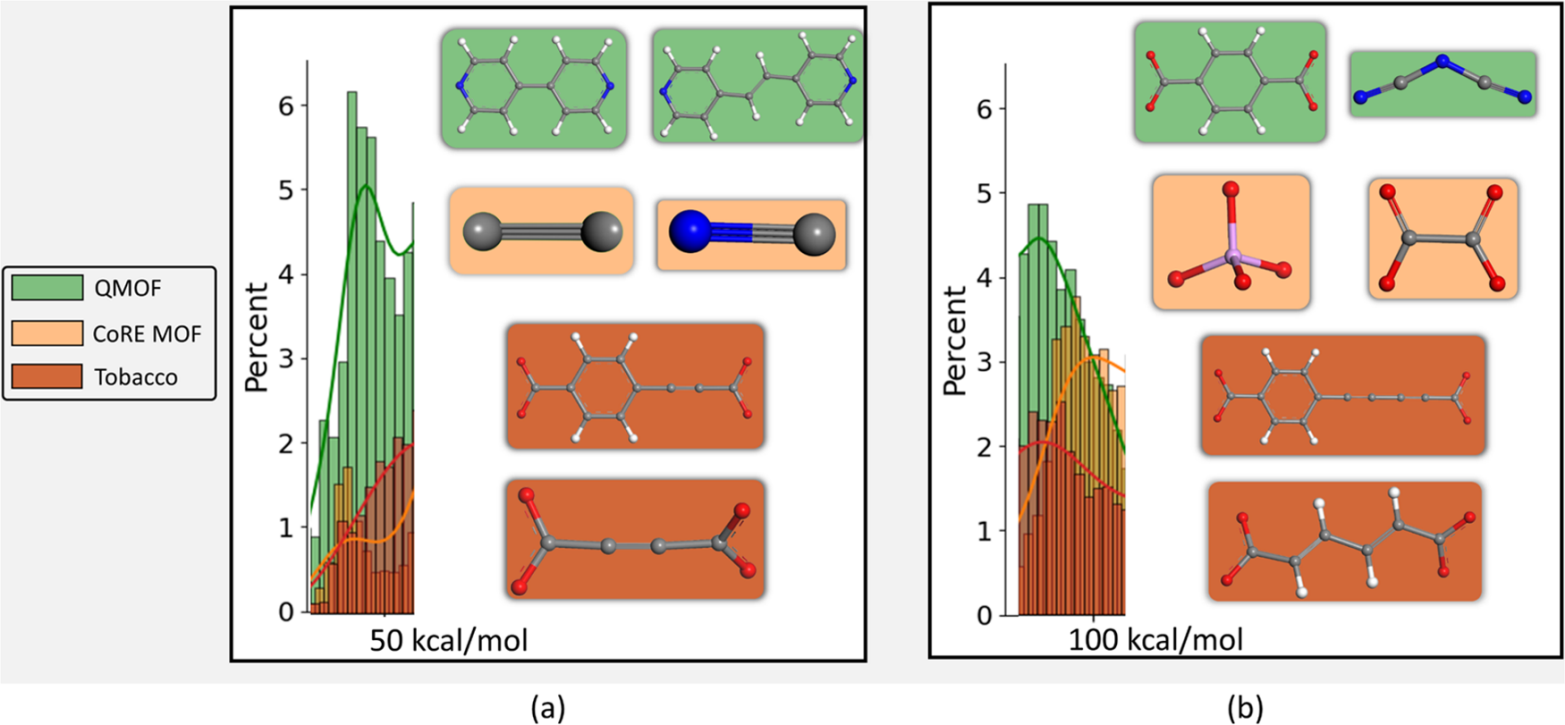
Two most frequent linkers found in energy ranges of (a) 0–50 and (b) 60–90 kcal/mol for each database.

By extracting data on linker concentration within specific energy ranges, valuable insights can be obtained regarding the most favorable linkers for stable MOF creation, enabling a reverse-engineering process. Focusing on the experimental databases reveals that the first distinct peak in the energy distribution for the QMOF and CoRE MOF is below the 50 kcal/mol mark. The most frequent linkers found in this region are depicted in Figure 8. In QMOF, two bipyridines emerge as the dominant linkers, whereas CoRE MOF predominantly features smaller ones, namely, one ethyne and one cyanide. QMOF’s bulkier linkers have lower-energy differences than CoRE’s, which can be attributed to the optimization process. Had they not been optimized, they would have lain in energy regions exceeding 120 kcal/mol, as observed by CoRE’s unoptimized MOF linkers which are formed by just a few atoms in these high-energy regions. QMOF’s unoptimized bulk linkers would have appeared impractical for synthesis. This underscores the pivotal role of optimization, which emerges as a crucial factor in enabling the realization of these MOFs. Regarding the hypothetical MOF database, we observe that the most common linkers in low-energy regions are benzene-1-carboxylate, 4-ethynecarboxylate, and ethyne dicarboxylate, indicating their potential as prime candidates for synthesizable MOFs. This workflow not only aids in identifying optimal linkers but also serves as a valuable tool for exploring the vast linker space to discern those that tend to generate synthesizable MOFs more frequently, thereby expanding the variety of synthesizable MOFs.

## Conclusions

In this study, we present a thorough exploration of the synthesizability likelihood in metal–organic framework databases such as QMOF, CoRE MOF, and ToBaCCo. We also developed a user-friendly tool that streamlines the computational process, offering accessibility to researchers with varying levels of computational expertise. Our findings highlight the importance of linker deformation in MOF synthesis, as it represents the energetic barriers that must be overcome for successful synthesis. We applied our methodology to over 40,000 MOFs, deconstructing them to analyze key parameters defining the linker strain within the MOF unit cell. We calculated single-point and optimization energies as well as root-mean-square deviation. Our results indicate that QMOF and CoRE MOF contain the most promising candidates for MOF synthesis, while ToBaCCo, composed of unoptimized materials, shows relatively poor synthesizability likelihood. Both findings align with our initial hypothesis based on the construction parameters of each database, which indicate that the experimental MOFs, particularly those optimized, would exhibit a higher synthesizability score. In addition, a reverse-engineering approach was employed by identifying optimal linker candidates that are frequently present among the best-performing MOFs, providing valuable guidance for experimental endeavors. Analysis of energy-distribution trends revealed a consistent pattern across databases, supported by high Pearson and Spearman coefficients exceeding 0.85. This suggests the possibility of omitting optimization calculations and reducing computational costs significantly. The computational tool developed in this study opens several promising avenues for future research in the field of metal–organic frameworks. By providing a reliable method for predicting the synthesizability of MOFs, researchers can more efficiently screen large databases of hypothetical MOFs, prioritizing those with a higher likelihood of successful synthesis. This approach significantly reduces the time and cost associated with experimental validation, making it a valuable asset for both academic research and industrial applications. The data generated from our tool can be used to train machine learning models that predict the MOF synthesizability with even greater accuracy. By leveraging artificial intelligence, we can enhance our predictive capabilities, making it possible to explore vast regions of the MOF space that is currently underexplored. While this study focuses on MOFs, the principles and computational methods developed here could be extended to other types of framework materials, such as covalent organic frameworks (COFs) and zeolitic imidazolate frameworks (ZIFs). This would allow for a broader application of the tool in the discovery of new materials with desirable properties. In conclusion, our comprehensive analysis of thousands of MOFs underscores the importance of linker deformation and energy disparities in understanding synthetic accessibility, offering valuable insights for advancing MOF research.

## Data Availability

The source code and example inputs and outputs are available at https://github.com/frudakis-research-group/mofsynth. QMOF database CIF files were fetched from the following link: https://github.com/Andrew-S-Rosen/QMOF. CoRE MOF latest version can be found at https://zenodo.org/records/7691378 and ToBaCCO database is available at https://github.com/tobacco-mofs/tobacco_1.0/tree/master. All generated files are available through request to the authors.
